# A State-Space Framework for Parallelizing Digital Signal Processing in Coherent Optical Receivers

**DOI:** 10.3390/s25237389

**Published:** 2025-12-04

**Authors:** Jinyang Wang, Zhugang Wang, Di Liu

**Affiliations:** 1 National Space Science Center, Chinese Academy of Sciences, Beijing 100190, China; wangjinyang21@mails.ucas.edu.cn (J.W.); liudi@nssc.ac.cn (D.L.); 2 School of Electromagnetic Field and Microwave Technology, University of Chinese Academy of Sciences, Beijing 100049, China

**Keywords:** parallel processing architectures, carrier synchronization, free-space optical communications, satellite optical communications

## Abstract

Ultra-high sampling rates in coherent optical front-ends increasingly exceed the processing capabilities of real-time baseband processors, creating a bottleneck in coherent free-space optical communication systems. We propose a unified state-space framework to systematically parallelize digital signal processing (DSP) algorithms. This approach transforms an algorithm’s transfer function into a state-space representation from which a parallel architecture is derived through matrix operations, overcoming the complexity of traditional ad hoc methods. Crucially, our framework enables an analysis of parallelization-induced latency. We introduce the parallel equivalent delay (PED) metric and demonstrate that it introduces right-half-plane zeros into the loop’s transfer function, thereby fundamentally constraining stability. This analysis leads to the derivation of “Throughput–Bandwidth Product” (TBP), a constant that provides a design guideline linking maximum stable loop bandwidth to the parallelization factor. The framework’s efficacy is demonstrated by designing a parallel Costas carrier recovery loop. Simulations validate its performance, confirm the TBP limit, and show significant advantages over conventional feedforward estimators, especially in low-SNR conditions. Implementation results on a AMD XCVU13P FPGA demonstrate that the proposed 50-parallel architecture achieves a throughput of 15.625 Gsps at a clock frequency of 312.5 MHz with a logic utilization below 7%. The experimental results confirm the theoretical trade-off between throughput and loop bandwidth, verifying the proposed design methodology.

## 1. Introduction

Coherent free-space optical communication (FSOC) has emerged as the preferred solution for high-speed, long-distance, inter-satellite links owing to its vast channel capacity, superior sensitivity, spectral efficiency, and interference resistance [1,2,3]. Although requiring sophisticated hardware components, such as narrow-linewidth lasers and high-performance processors that support real-time digital signal processing, its performance benefits are essential in mission-critical scenarios, including deep-space exploration and high-capacity inter-satellite data transmission. This trend has been supported by rapid advancements in high-speed data converters, with analog-to-digital (ADC) and digital-to-analog (DAC) devices now achieving sampling rates in the tens of giga-samples per second (GSPS), providing a robust hardware foundation for ultra-high-speed transceivers [4]. However, a major bottleneck persists between the front-end sampling rate and the back-end digital signal processing (DSP) capability [5]. The clock frequencies of contemporary baseband processors, such as field-programmable gate arrays (FPGAs) and digital signal processors, lag far behind these ultra-high sampling rates. To enable true real-time operation, parallel processing architectures must be adopted [6]. By decomposing a high-speed data stream into multiple lower-speed parallel streams, DSP algorithms can be executed on baseband processors operating at feasible clock rates.

In high-speed coherent baseband DSP, parallelization techniques generally fall into three main categories: (i) Polyphase decomposition-based architectures: Widely used for parallelizing finite impulse response (FIR) filters, this method partitions filter coefficients into multiple subfilters operating on decimated input streams, then recombines the outputs. When combined with coefficient symmetry, it reduces per-branch computational load and enables efficient hardware mapping [7]. (ii) Look-ahead and buffered memory structures: Buffered memory schemes exploit pipelining and interleaving to maintain continuous operation across parallel streams. Look-ahead transformations are often applied to recursive filters to mitigate feedback latency while sustaining throughput [8]. This approach is also effective for FIR filters via distributed parallel structures [9]. However, in high-order feedback loops such as phase-locked loops (PLLs), maintaining exact serial equivalence at high parallelism factors increases design complexity sharply due to strong dependence on loop order and parallelism degree. (iii) Frequency-domain parallel computing: This method transforms time-domain DSP operations into the frequency domain using the fast Fourier transform (FFT), executes parallel computations, and then employs the inverse FFT (IFFT) to restore the time-domain signal. It is particularly advantageous for block-based algorithms, including certain equalizers and synchronization schemes.

Parallel architectures for coherent receivers can be classified into three categories: open-loop (feedforward), closed-loop (feedback), and hybrid systems. (i) Open-loop (feedforward) parallel receivers: Feedforward architectures are inherently suited for parallelization as they operate on blocks of data without recursive dependencies. This structure allows for deep pipelining in hardware (FPGA/ASIC) implementations, making them a popular choice for ultra-high-speed systems [10,11]. Classic blind feedforward algorithms, such as the Viterbi–Viterbi (VV) and Blind Phase Search (BPS) methods, estimate carrier phase and frequency offset by processing a block of received symbols at once [12,13,14]. While effective, their performance can be sensitive to block size, and they may struggle with very large frequency offsets or rapid phase fluctuations. To enhance robustness, data-aided or pilot-aided feedforward schemes are often employed, particularly for initial wide-range frequency acquisition [15,16,17,18]. (ii) Closed-loop (feedback) parallel receivers: Typically based on PLLs, these architectures excel at tracking time-varying impairments such as laser phase noise or frequency drift. However, their recursive nature, where each symbol’s processing depends on previous results, creates a “feedback bottleneck” for parallelization. Several strategies exist: (a) Polyphase-based architectures: One classical approach is to use look-ahead transformations, often realized through polyphase decomposition. This receiver architecture is called a parallel receiver (PRX) [19]. This technique mathematically unfolds the serial loop recursion into a set of parallel equations, allowing multiple output symbols to be computed simultaneously. While this creates a structurally parallel system, it often results in a significant increase in computational complexity per symbol processed [20]. (b) Frequency-domain transform: A frequency-domain closed-loop architecture is known as Alternative Parallel Receiver (APRX), which leverages the properties of the fast Fourier transform (FFT) [21]. By transforming a block of signals into the frequency domain, the time-domain convolution within the feedback loop becomes a simple multiplication. Architectures like the APRX perform loop filtering and correction in the frequency domain before transforming the signal back to the time domain [22,23,24]. This approach enables efficient block-based parallel processing. (c) Multi-stream averaging or simplified updates: These approximate schemes average error signals from all streams to produce a common correction term [25], reducing complexity but slightly degrading tracking accuracy. (d) Buffered structures with serial equivalence: Designs aiming for exact equivalence with serial loops rely heavily on look-ahead derivations [26]. Although theoretically capable under arbitrary parallelism, complexity grows rapidly with the loop order and parallelism factor, limiting practical use for high-order PLLs. (iii) Hybrid parallel receivers: These architectures combine feedforward and feedback stages to leverage both wide capture range and fine tracking [12,16,27]. A common approach is to use a feedforward stage for coarse frequency/phase estimation, followed by a parallelized feedback loop for precise tracking, thus balancing acquisition speed and steady-state performance.

Despite these diverse approaches, achieving high tracking accuracy, low complexity, and minimal latency in a parallel carrier-recovery system remains an open challenge. Ideally, such a system would offer the robustness of feedback loops and the architectural simplicity of feedforward methods.

Motivated by this goal, we propose a state-space-based parallelization framework that unifies the design of both open-loop and closed-loop DSP algorithms. The main contributions of this work are as follows: (i) We propose a unified state-space modeling framework that automatically maps a broad class of serial DSP algorithms (FIR/IIR, PLL-based carrier recovery, etc.) into parallel multi-input multi-output architectures using only matrix operations. Unlike prior look-ahead and polyphase-based derivations, the proposed procedure is independent of loop order and parallelism degree, and therefore scales gracefully to high-order, high-parallelism feedback systems. (ii) Beyond structural parallelization, we introduce the notion of parallel equivalent delay (PED), which explicitly captures both structural and computational latency in state-space-based parallel feedback loops. We show analytically that PED induces right-half-plane zeros in the loop transfer function, a phenomenon not treated in earlier parallelization-oriented works. (iii) Based on this delay model, we derive a Throughput–Bandwidth Product (TBP) constraint, which links achievable loop bandwidth to implementation-level delay. (iv) We validate the framework through both numerical simulation and FPGA implementation (50-way parallel, 15.625 Gsps), and empirically confirm the predicted TBP behavior.

The remainder of this paper is organized as follows: Section 2 introduces the state-space framework and details the systematic procedure for parallelizing any FIR filter. In Section 3, we apply this framework to a practical example, deriving a highly parallel structure for the second-order Costas loop. Section 4 presents an analysis of the stability of parallel feedback systems, introducing the concept of parallel equivalent delay (PED) and deriving the fundamental Throughput–Bandwidth Product limit. In Section 5, we provide simulation results to validate the proposed framework, verify our theoretical analysis, and compare its performance against conventional feedforward algorithms. Finally, Section 6 presents the hardware implementation results on a XCVU13P FPGA (AMD, San Jose, CA, USA). We demonstrate a 50-parallel design achieving 15.625 Gsps and empirically verify the theoretical TBP trade-off under actual timing constraints. Section 7 concludes the paper.

## 2. The State-Space Parallelization

### 2.1. General Theory of State-Space Parallelization and Related Work

Discrete-time linear signal processing algorithms, encompassing both feedforward (FIR) and feedback (IIR) architectures, can be universally described using the state-space representation. The theoretical foundation for parallelizing these structures was established by Parhi et al. [28] and further explored on SIMD architectures by Robelly et al. [29]. While these works focus on arithmetic transformations, this paper employs the state-space framework to analyze the stability boundaries of high-speed parallel systems.

State-space equations characterize a system in the time domain through a set of first-order differential equations, establishing a mapping between the system’s internal state variables, external inputs, and outputs in matrix form. For discrete systems, the state-space representation is expressed as:

(1) $$\begin{array}{cc} x(k+1) & =Ax(k)+Bu(k) \end{array}$$

(2) $$\begin{array}{cc} y(k) & =Cx(k)+Du(k) \end{array}$$

where x(k) denotes the state vector, representing the internal state of the system; x(k+1) denotes the next state vector; u(k) signifies the system input; and y(k) represents the system output. Matrix A, the state matrix, governs the evolution of the internal states; B, the input matrix, describes the influence of the input on the system states; C, the output matrix, provides a linear mapping from the internal states to the output; and D, the feedforward matrix, captures the direct effect of the input on the output.

Equation (1) represents the state transition equation, describing the relationship between the next state, the current state, and the current input, while Equation (2) is the output equation, describing the relationship between the system output, the current state, and the current input. To parallelize this representation with a parallelism factor *N*, we define batched input and output vectors over N-step horizons:

(3) $$\bar{u}\left(k\right)=\left[\begin{array}{c} u(Nk)\\ u(Nk+1)\\ \vdots \\ u(Nk+N-1) \end{array}\right]\bar{y}\left(k\right)=\left[\begin{array}{c} y(Nk)\\ y(Nk+1)\\ \vdots \\ y(Nk+N-1) \end{array}\right]$$

The state evolution can then be expressed as:

(4) $$\begin{array}{cc} x(Nk+1) & =Ax(Nk)+Bu(Nk)\\ x(Nk+2) & =A^{2}x\left(Nk\right)+\mathrm{AB}u\left(Nk\right)+Bu\left(Nk+1\right)\\ \; & \vdots \\ x(N(k+1)) & =A^{N}x\left(Nk\right)+A^{N-1}Bu\left(Nk\right)\\ \; & +\;A^{N-2}Bu\left(Nk+1\right)+\cdots \\ \; & +\;Bu(Nk+N-1) \end{array}$$

Similarly, the output equations are derived as:

(5) $$\begin{array}{c} y(Nk)=Cx(Nk)+Du(Nk)\\ y(Nk+1)=\mathrm{CA}x(Nk)+\mathrm{CB}u(Nk)+Du(Nk+1)\\ y\left(Nk+2\right)={\mathrm{CA}}^{2}x\left(Nk\right)+\mathrm{CAB}u\left(Nk\right)+\mathrm{CB}u\left(Nk+1\right)\\ +Du(Nk+2)\\ \left[\begin{array}{c} y(Nk)\\ y(Nk+1)\\ \vdots \\ y(Nk+N-1) \end{array}\right]=\left[\begin{array}{c} C\\ \mathrm{CA}\\ \vdots \\ {\mathrm{CA}}^{N-1} \end{array}\right]x\left(Nk\right)\\ +\left[\begin{array}{cccc} D & 0 & \ldots & 0\\ \mathrm{CB} & D & \ldots & 0\\ \vdots & \vdots & \ddots & \vdots \\ {\mathrm{CA}}^{N-2}B & {\mathrm{CA}}^{N-3}B & \ldots & D \end{array}\right]\left[\begin{array}{c} u(Nk)\\ u(Nk+1)\\ \vdots \\ u(Nk+N-1) \end{array}\right] \end{array}$$

Thus, the parallelized state-space representation of the original equations is:

(6) $$\begin{array}{c} \bar{x}\left(k+1\right)=\bar{A}\bar{x}\left(k\right)+\bar{B}\bar{u}\left(k\right)\\ \bar{y}\left(k\right)=\bar{C}\bar{x}\left(k\right)+\bar{D}\bar{u}\left(k\right) \end{array}$$

where

(7) $$\begin{array}{cc} \; & \bar{x}\left(k\right)=x\left(Nk\right) \end{array}$$

(8) $$\begin{array}{cc} \; & \bar{A}=A^{N} \end{array}$$

(9) $$\begin{array}{cc} \; & \bar{B}=\left[A^{N-1}B\;A^{N-2}B\;\ldots \;\mathrm{AB}\;B\right] \end{array}$$

(10) $$\begin{array}{cc} \; & \bar{C}=\left[\begin{array}{c} C\\ \mathrm{CA}\\ \vdots \\ {\mathrm{CA}}^{N-1} \end{array}\right] \end{array}$$

(11) $$\begin{array}{cc} \; & \bar{D}=\left[\begin{array}{cccc} D & 0 & \ldots & 0\\ \mathrm{CB} & D & \ldots & 0\\ \vdots & \vdots & \ddots & \vdots \\ {\mathrm{CA}}^{N-2}B & {\mathrm{CA}}^{N-3}B & \ldots & D \end{array}\right] \end{array}$$

This represents the parallelized form of the original state-space equations.

The standard form of the transfer function for a digital filter is expressed as follows:

(12) $$\begin{array}{cc} H\left(z\right) & =\frac{Y\left(z\right)}{X\left(z\right)} \end{array}$$

(13) $$\begin{array}{cc} \; & =\frac{b_{0}+b_{1}z^{-1}+\cdots +b_{n-1}z^{-\left(n-1\right)}+b_{n}z^{-n}}{1+a_{1}z^{-1}+\cdots +a_{n-1}z^{-\left(n-1\right)}+a_{n}z^{-n}} \end{array}$$

To convert this transfer function into a state-space representation, we first transform it into a differential equation by cross-multiplication:

(14) $$\begin{array}{cc} Y\left(z\right)(1+a_{1}z^{-1} & +\cdots a_{n}z^{-n}) \end{array}$$

(15) $$\begin{array}{cc} \; & =X\left(z\right)\left(b_{0}+b_{1}z^{-1}+\cdots +b_{n}z^{-n}\right) \end{array}$$

Rearranging the terms for Y(z) yields:

(16) $$\begin{array}{cc} Y(z) & =-a_{1}z^{-1}Y\left(z\right)-\cdots -a_{n}z^{-n}Y\left(z\right)\\ \; & +b_{0}X\left(z\right)+b_{1}z^{-1}X\left(z\right)+\cdots +b_{n}z^{-n}X\left(z\right) \end{array}$$

Taking the inverse Z-transform, the corresponding time-domain differential equation is:

(17) $$\begin{array}{cc} y(k) & =-a_{1}y\left(k-1\right)-\cdots -a_{n}y\left(k-n\right)+b_{0}x\left(k\right)\\ \; & +b_{1}x\left(k-1\right)+\cdots +b_{n}x\left(k-n\right) \end{array}$$

For the derivation of the state-space representation, an auxiliary variable W(z) is introduced such that:

(18) $$H\left(z\right)=\frac{Y(z)}{X(z)}=\frac{Y(z)}{W(z)}\cdot \frac{W(z)}{X(z)}$$

Specifically, we define:

(19) $$\frac{W(z)}{X(z)}=\frac{1}{1+a_{1}z^{-1}+\cdots +a_{n}z^{-n}}$$

and

(20) $$\frac{Y(z)}{W(z)}=b_{0}+b_{1}z^{-1}+\cdots +b_{n}z^{-n}$$

Consequently, in the time domain, we obtain the following differential equations:

(21) $$w\left(k\right)=x\left(k\right)-a_{1}w\left(k-1\right)-\cdots -a_{n}w\left(k-n\right)$$

The output equation is:

(22) $$y\left(k\right)=b_{0}w\left(k\right)+b_{1}w\left(k-1\right)+\cdots +b_{n}w\left(k-n\right)$$

The state variables are then defined as:

(23) $$\begin{array}{cc} \; & x_{1}\left(k\right)=w\left(k-n\right)\\ \; & x_{2}\left(k\right)=w\left(k-n+1\right)\\ \; & \vdots \\ \; & x_{n}\left(k\right)=w\left(k-1\right) \end{array}$$

The state update equations are derived as:

(24) $$\begin{array}{cc} \; & x_{1}\left(k+1\right)=x_{2}\left(k\right)\\ \; & x_{2}\left(k+1\right)=x_{3}\left(k\right)\\ \; & \vdots \\ \; & x_{n-1}\left(k+1\right)=x_{n}\left(k\right)\\ \; & x_{n}\left(k+1\right)=w\left(k\right)\\ \; & =x\left(k\right)-a_{1}w\left(k-1\right)-a_{2}w\left(k-2\right)-\cdots -a_{n}w\left(k-n\right) \end{array}$$

Substituting the definitions of the state variables into the equation for $x_{n}\left(k+1\right)$ yields:

(25) $$x_{n}\left(k+1\right)=-a_{n}x_{1}\left(k\right)-a_{n-1}x_{2}\left(k\right)-\cdots -a_{1}x_{n}\left(k\right)+x\left(k\right)$$

From this, the state matrix A is determined as:

(26) $$A=\left[\begin{array}{ccccc} 0 & 1 & 0 & \ldots & 0\\ 0 & 0 & 1 & \ldots & 0\\ \vdots & \vdots & \vdots & \ddots & \vdots \\ 0 & 0 & 0 & \ldots & 1\\ -a_{n} & -a_{n-1} & -a_{n-2} & \ldots & -a_{1} \end{array}\right]$$

The input matrix B is given as:

(27) $$B=\left[\begin{array}{c} 0\\ 0\\ \vdots \\ 0\\ 1 \end{array}\right]$$

For the output equation, we express $w\left(k\right)$ and its delayed versions in terms of the defined state variables:

(28) $$\begin{array}{cc} \; & w\left(k\right)=x\left(k\right)-a_{1}x_{n}\left(k\right)-a_{2}x_{n-1}\left(k\right)-\cdots -a_{n}x_{1}\left(k\right)\\ \; & w\left(k-1\right)=x_{n}\left(k\right)\\ \; & w\left(k-2\right)=x_{n-1}\left(k\right)\\ \; & \;\;\vdots \\ \; & w\left(k-n\right)=x_{1}\left(k\right) \end{array}$$

Substituting these expressions into the output equation for $y\left(k\right)$:

(29) $$\begin{array}{cc} y\left(k\right)=b_{0}(x\left(k\right) & -\;a_{1}x_{n}\left(k\right)-\cdots -a_{n}x_{1}\left(k\right))+b_{1}x_{n}\left(k\right)\\ \; & +\;b_{2}x_{n-1}\left(k\right)+\cdots +b_{m}x_{1}\left(k\right) \end{array}$$

Collecting terms, we obtain:

(30) $$\begin{array}{cc} y\left(k\right)=\left(b_{n}-a_{n}b_{0}\right)x_{1}\left(k\right) & +\;\cdots +\left(b_{1}-a_{1}b_{0}\right)x_{n}\left(k\right)\\ \; & +\;b_{0}x\left(k\right) \end{array}$$

Consequently, the output matrix C is given as:

(31) $$C=\left[\begin{array}{cccc} \left(b_{n}-a_{n}b_{0}\right) & \left(b_{n-1}-a_{n-1}b_{0}\right) & \ldots & \left(b_{1}-a_{1}b_{0}\right) \end{array}\right]$$

The direct transmission matrix D is given as:

(32) $$D=\left[b_{0}\right]$$

In summary, the state-space representation is given by:

(33) $$x\left(k+1\right)=\left[\begin{array}{ccccc} 0 & 1 & 0 & \ldots & 0\\ 0 & 0 & 1 & \ldots & 0\\ \vdots & \vdots & \vdots & \ddots & \vdots \\ 0 & 0 & 0 & \ldots & 1\\ -a_{n} & -a_{n-1} & -a_{n-2} & \ldots & -a_{1} \end{array}\right]x\left(k\right)+\left[\begin{array}{c} 0\\ 0\\ \vdots \\ 0\\ 1 \end{array}\right]u\left(k\right)$$

(34) $$y\left(k\right)={\left[\begin{array}{c} \left(b_{n}-a_{n}b_{0}\right)\\ \left(b_{n-1}-a_{n-1}b_{0}\right)\\ \ldots \\ \left(b_{1}-a_{1}b_{0}\right) \end{array}\right]}^{T}x\left(k\right)+\left[b_{0}\right]u\left(k\right)$$

Once the serial filter’s state-space matrices (A,B,C,D) are defined, the parallel system matrices $\left(\bar{A},\bar{B},\bar{C},\bar{D}\right)$ are derived using Equations (8)–(11), with the parallel state defined in Equation (7).

### 2.2. Application to Feedforward Systems: The Parallel FIR Filter

The finite impulse response (FIR) filter is a fundamental component in digital communication systems, often used as a matched or pulse-shaping filter for optimal signal detection. We will demonstrate the process to parallelize an FIR filter. An M-tap serial FIR filter is described by the differential equation:

(35) $$y\left(k\right)=b_{0}x\left(k\right)+b_{1}x\left(k-1\right)+\cdots +b_{M}x\left(k-M\right)$$

Its transfer function is:

(36) $$H\left(z\right)=b_{0}+b_{1}z^{-1}+\cdots +b_{M}z^{-M}$$

This is a special case of the general transfer function (13) where all denominator coefficients $a_{i}$ (for i≥1) are zero. Following the derivation procedure in Section 2, we can represent an M-tap FIR filter in state-space form:

(37) $$\begin{array}{c} x\left(k+1\right)=\underset{A}{\underset{︸}{\left[\begin{array}{ccccc} 0 & 1 & 0 & \ldots & 0\\ 0 & 0 & 1 & \ldots & 0\\ \vdots & \vdots & \vdots & \ddots & \vdots \\ 0 & 0 & 0 & \ldots & 1\\ 0 & 0 & 0 & \ldots & 0 \end{array}\right]}}x\left(k\right)+\underset{B}{\underset{︸}{\left[\begin{array}{c} 0\\ 0\\ \vdots \\ 0\\ 1 \end{array}\right]}}u\left(k\right)\\ y\left(k\right)=\underset{C}{\underset{︸}{\left[\begin{array}{cccc} b_{M} & b_{M-1} & \ldots & b_{1} \end{array}\right]}}x\left(k\right)+\underset{D}{\underset{︸}{\left[\begin{array}{c} b_{0} \end{array}\right]}}u\left(k\right) \end{array}$$

With the serial FIR filter represented by matrices A,B,C,D, we can directly apply the parallel decomposition Equations (8) and (11) to obtain the parallel MIMO system matrices $\bar{A},\bar{B},\bar{C},\bar{D}$ with a parallelism factor N. The parallel FIR filter then processes *N* input samples $\bar{u}\left(k\right)={\left[u\left(Nk\right)\ldots u\left(Nk+N-1\right)\right]}^{T}$ simultaneously to produce *N* output samples $\bar{y}\left(k\right)={\left[y\left(Nk\right)\ldots y\left(Nk+N-1\right)\right]}^{T}$ using:

(38) $$\begin{array}{c} \bar{x}\left(k+1\right)=\bar{A}\bar{x}\left(k\right)+\bar{B}\bar{u}\left(k\right)\\ \bar{y}\left(k\right)=\bar{C}\bar{x}\left(k\right)+\bar{D}\bar{u}\left(k\right) \end{array}$$

where $\bar{x}\left(k\right)$ represents the state vector $x\left(Nk\right)$.

Since the eigenvalues of A are zero (or the system is open-loop), the latency introduced by parallelization (pipelining) only delays the output data stream but does not affect the system’s transfer function or stability. This represents the simplest case of parallelization.

## 3. Application to Feedback Systems: The Parallel Costas Loop

**Figure 1:**
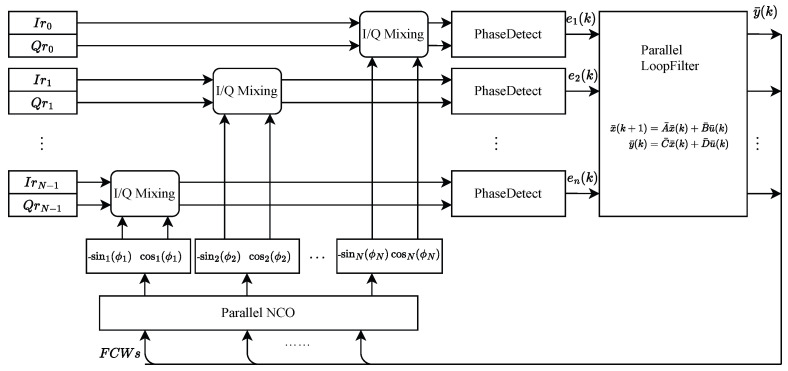
Parallel implementation of the Costas loop for QPSK demodulation.

This paper presents a parallel implementation of the Costas loop for QPSK demodulation, as illustrated in Figure 1. The Costas loop comprises a quadrature mixer, a phase detector, a loop filter, and a numerically controlled oscillator (NCO). Among these components, the quadrature mixers and phase detectors, being memoryless, are parallelized by simple replication across *N* channels, with each channel n operating as:

(39) $$\begin{array}{c} \left\{\begin{array}{c} I_{n}\left(k\right)=Ir_{n}\left(k\right)\cdot \cos_{n}\left(\phi_{n}\right)+Qr_{n}\left(k\right)\cdot \sin_{n}\left(\phi_{n}\right)\\ Q_{n}\left(k\right)=Qr_{n}\left(k\right)\cdot \cos_{n}\left(\phi_{n}\right)-Ir_{n}\left(k\right)\cdot \sin_{n}\left(\phi_{n}\right) \end{array}\right. \end{array}$$

(40) $$\begin{array}{c} e_{n}\left(k\right)=Q_{n}\left(k\right)\cdot \mathrm{sign}\left(I_{n}\left(k\right)\right)-I_{n}\left(k\right)\cdot \mathrm{sign}\left(Q_{n}\left(k\right)\right) \end{array}$$

### 3.1. Parallel NCO

A numerically controlled oscillator (NCO) is primarily composed of three key components: a phase accumulator, a phase register, and a Look-Up Table (LUT). The phase accumulator, driven by the sampling clock, performs discrete-time integration through incremental summation of the frequency control word (FCW), effectively functioning as an integrator. The bit width of the phase accumulator is denoted by W, and it automatically wraps around upon overflow, which can be seen as performing a modulo operation with $2^{W}$ on the integration result. The phase register stores the accumulated phase value, which serves as the address input to the LUT. The LUT, in turn, establishes a mapping between the input address and the corresponding output amplitude value.

For parallel implementation, both the modulo operation (inherent in the accumulator’s wrap-around) and the LUT can be readily realized by simple replication across multiple processing paths. When parallelizing the integrator component, its transfer function is considered to be:

(41) $$\frac{1}{1-z^{-1}}$$

For this system, the discrete-time state-space matrices are:

(42) $$\begin{array}{c} A=\left[-a_{1}\right]=\left[1\right]\\ B=[1]\\ C=\left[b_{1}-a_{1}b_{0}\right]=\left[1\right]\\ D=\left[b_{0}\right]=\left[1\right] \end{array}$$

The corresponding state-space equations are:

(43) $$\left\{\begin{array}{c} x(k+1)=x(k)+u(k)\\ y(k)=x(k)+u(k) \end{array}\right.$$

From (8) to (11), the matrices for an N-parallel system $\left(\bar{A},\bar{B},\bar{C},\bar{D}\right)$ can be derived as follows:

(44) $$\begin{array}{cc} \; & \bar{A}=A^{N}=\left[1\right]\\ \; & \bar{B}=\left[A^{N-1}B\;A^{N-2}B\;\ldots \;\mathrm{AB}\;B\right]=\left[1\;1\;\ldots \;1\right]\\ \; & \bar{C}=\left[\begin{array}{c} C\\ \mathrm{CA}\\ \vdots \\ {\mathrm{CA}}^{N-1} \end{array}\right]=\left[\begin{array}{c} 1\\ 1\\ \vdots \\ 1 \end{array}\right]\\ \; & \bar{D}=\left[\begin{array}{cccc} D & 0 & \ldots & 0\\ \mathrm{CB} & D & \ldots & 0\\ \vdots & \vdots & \ddots & \vdots \\ {\mathrm{CA}}^{N-2}B & {\mathrm{CA}}^{N-3}B & \ldots & D \end{array}\right]=\left[\begin{array}{cccc} 1 & 0 & \ldots & 0\\ 1 & 1 & \ldots & 0\\ \vdots & \vdots & \ddots & \vdots \\ 1 & 1 & \ldots & 1 \end{array}\right] \end{array}$$

The N-channel parallelized state-space equations are then:

(45) $$\begin{array}{c} \bar{x}\left(k+1\right)=\bar{x}\left(k\right)+\left[\begin{array}{cccc} 1 & 1 & \ldots & 1 \end{array}\right]\bar{u}\left(k\right)\\ \bar{y}\left(k\right)=\left[\begin{array}{c} 1\\ 1\\ \vdots \\ 1 \end{array}\right]\bar{x}\left(k\right)+\left[\begin{array}{cccc} 1 & 0 & \ldots & 0\\ 1 & 1 & \ldots & 0\\ \vdots & \vdots & \ddots & \vdots \\ 1 & 1 & \ldots & 1 \end{array}\right]\bar{u}\left(k\right) \end{array}$$

**Figure 2:**
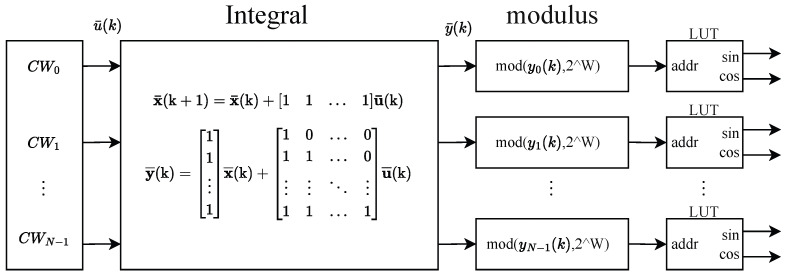
Parallel implementation of NCO.

As depicted in Figure 2, these components are combined to achieve an N-path parallel NCO implementation.

### 3.2. Parallel Loop Filter

The loop filter of a second-order phase-locked loop (PLL) is a first-order infinite impulse response (IIR) filter, Commonly, a Proportional–Integral (PI) structure is employed for this purpose. Its discrete-time transfer function, H(z), is given by:

(46) $$\begin{array}{c} H\left(z\right)=K_{p}+K_{i}\frac{T}{1-z^{-1}} \end{array}$$

where $K_{p}$ and $K_{i}$ represent the proportional gain and integral gain, respectively, and *T* denotes the sampling period.

**Figure 3:**
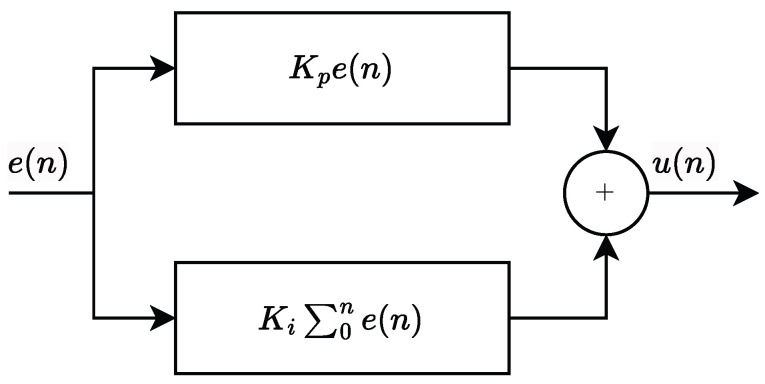
The structure of Proportional–Integral filter.

As shown in Figure 3, the operation of the PI filter involves two components acting on the phase error signal, e(n). The proportional term, $K_{p}\cdot e\left(n\right)$, provides an instantaneous control action based on the current error. The integral term, $K_{i}T\cdot \sum_{k=0}^{n}e\left(k\right)$, accumulates the historical error, effectively integrating the phase error over time. The total control output signal, $u\left(n\right)$, is the superposition of these two terms.

From a control perspective, increasing the proportional gain ($K_{p}$) generally enhances the loop’s response speed. However, excessively high values can lead to undesirable overshoot or sustained oscillations in the transient response. The integral gain ($K_{i}$) is primarily responsible for eliminating steady-state phase error, thereby ensuring phase lock accuracy. Nonetheless, an overly large $K_{i}$ can detrimentally affect loop stability margins.

In the design process of digital PLLs (DPLLs), the gains $K_{p}$ and $K_{i}$ are typically determined based on the target loop noise bandwidth ($B_{L}$). This parameter is crucial for achieving the desired trade-off between system stability and dynamic performance characteristics. $B_{L}$ quantifies the loop’s susceptibility to input noise; specifically, a narrower bandwidth (smaller $B_{L}$) improves the rejection of high-frequency noise components but consequently results in a slower dynamic response. For a standard second-order type-II PLL, the loop noise bandwidth is related to the loop’s natural angular frequency ($\omega_{n}$) and damping factor (ζ) via the expression:

(47) $$\begin{array}{c} B_{L}=\frac{\omega_{n}}{2}\left(\zeta +\frac{1}{4\zeta }\right) \end{array}$$

This relationship allows for the determination of the required natural angular frequency based on the specified $B_{L}$ and chosen ζ:

(48) $$\begin{array}{c} \omega_{n}=\frac{2B_{L}}{\zeta +\frac{1}{4\zeta }} \end{array}$$

Subsequently, the proportional and integral gains can be calculated using the following formulae, which incorporate the phase detector gain ($K_{pd}$) and the oscillator gain ($K_{o}$):

(49) $$K_{p}\;=\;\frac{2\zeta \omega_{n}}{K_{d}K_{o}}\;,\;K_{i}=\frac{\omega_{n}^{2}}{K_{d}K_{o}}$$

The damping factor, ζ, is frequently selected to be approximately 0.707, corresponding to critical damping or an optimal balance between settling time and overshoot.

An alternative representation of the PI filter’s transfer function $H\left(z\right)=Y\left(z\right)/U\left(z\right)$, where $Y\left(z\right)$ is the output and $U\left(z\right)$ is the input in the z-domain, can be obtained by defining coefficients $C_{1}=K_{p}+K_{i}T$ and $C_{2}=-K_{p}$. This yields:

(50) $$\begin{array}{c} H\left(z\right)=\frac{C_{1}+C_{2}z^{-1}}{1-z^{-1}} \end{array}$$

This form is often convenient for digital implementation. The corresponding state-space representation of this filter is expressed as:

(51) $$\left\{\begin{array}{c} x(k+1)=[1]x(k)+[1]u(k)\\ y\left(k\right)=\left[C_{1}+C_{2}\right]x\left(k\right)+\left[C_{1}\right]u\left(k\right) \end{array}\right.$$

Based on Equations (8)–(11),

(52) $$\begin{array}{cc} \; & \bar{A}=A^{N}=\left[1\right] \end{array}$$

(53) $$\begin{array}{cc} \; & \bar{B}=\left[\begin{array}{ccccc} A^{N-1}B & A^{N-2}B & \ldots & \mathrm{AB} & B \end{array}\right]=\left[\begin{array}{cccc} 1 & 1 & \ldots & 1 \end{array}\right] \end{array}$$

(54) $$\begin{array}{cc} \; & \bar{C}=\left[\begin{array}{c} C\\ \mathrm{CA}\\ \vdots \\ {\mathrm{CA}}^{N-1} \end{array}\right]=\left[\begin{array}{c} C_{1}+C_{2}\\ C_{1}+C_{2}\\ \vdots \\ C_{1}+C_{2} \end{array}\right] \end{array}$$

(55) $$\begin{array}{c} \bar{D}=\left[\begin{array}{cccc} D & 0 & \ldots & 0\\ \mathrm{CB} & D & \ldots & 0\\ \vdots & \vdots & \ddots & \vdots \\ {\mathrm{CA}}^{N-2}B & {\mathrm{CA}}^{N-3}B & \ldots & D \end{array}\right]\\ =\left[\begin{array}{cccc} C_{1} & 0 & \ldots & 0\\ C_{1}+C_{2} & C_{1} & \ldots & 0\\ \vdots & \vdots & \ddots & \vdots \\ C_{1}+C_{2} & C_{1}+C_{2} & \ldots & C_{1} \end{array}\right] \end{array}$$

The parallelized expression derived from the state-space equations can be formulated as follows:

(56) $$\begin{array}{c} \bar{x}\left(k+1\right)=\left[1\right]\bar{x}\left(k\right)+\left[1\;1\;\ldots \;1\right]\bar{u}\left(k\right)\\ \bar{y}\left(k\right)=\left[\begin{array}{c} C_{1}+C_{2}\\ C_{1}+C_{2}\\ \vdots \\ C_{1}+C_{2} \end{array}\right]\bar{x}\left(k\right)+\left[\begin{array}{cccc} C_{1} & 0 & \ldots & 0\\ C_{1}+C_{2} & C_{1} & \ldots & 0\\ \vdots & \vdots & \ddots & \vdots \\ C_{1}+C_{2} & C_{1}+C_{2} & \ldots & C_{1} \end{array}\right]\bar{u}\left(k\right) \end{array}$$

## 4. Stability Analysis and Design Methodology for Parallel Feedback Loops

While the state-space framework guarantees mathematical equivalence between the serial and parallel implementations, the “parallel equivalent delay” (PED) fundamentally alters the loop characteristics compared to its serial counterpart. In this section, we first analyze the origin and impact of PED within the context of our state-space parallelized PLL, then derive a predictive design guideline that links system throughput, hardware implementation, and maximum achievable loop bandwidth. Finally, we reveal a fundamental trade-off, the “Throughput–Bandwidth Product”, which governs the performance limits of such parallel feedback systems.

### 4.1. Costas Loop Model and Normalization

For a standard, serial, second-order phase-locked loop (PLL) employing a Proportional–Integral (PI) loop filter, the open-loop transfer function $G_{o}\left(s\right)$ is given by:

(57) $$G_{o}\left(s\right)=K_{pd}\cdot F\left(s\right)\cdot \frac{K_{o}}{s}$$

where $K_{pd}$ is the phase detector gain, $K_{o}$ is the oscillator gain, and $F\left(s\right)=K_{p}+K_{i}/s=K_{p}\left(s+K_{i}/K_{p}\right)/s$ is the PI filter’s transfer function. Substituting F(s) yields:

(58) $$G_{o}\left(s\right)=K_{pd}\cdot K_{p}\cdot K_{o}\cdot \frac{s+z}{s^{2}}=K\frac{s+z}{s^{2}}$$

**Figure 4:**
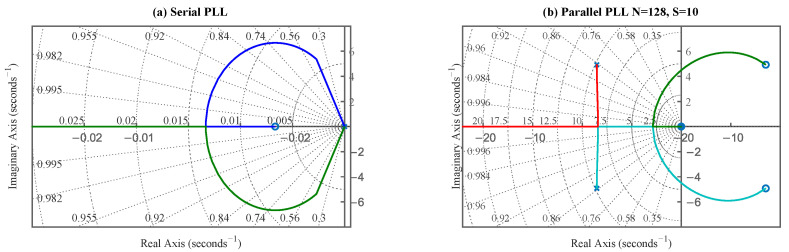
(**a**) Root locus of the ideal serial PLL, demonstrating unconditional stability. (**b**) Root locus of the parallel PLL, showing stability reduction due to the RHP zero introduced by PED.

Here, $K=K_{pd}\cdot K_{p}\cdot K_{o}$ represents the total loop gain, and $z=K_{i}/K_{p}$. The system exhibits a double pole at s=0 and a single zero at s=−z. Root locus analysis, as shown in Figure 4a, reveals that for a stable filter (z>0), the branches remain entirely within the left-half-plane (LHP) for all positive loop gains (K>0). This demonstrates that the idealized second-order serial PLL is unconditionally stable, with loop gain K primarily affecting damping characteristics.

The considered demodulator is based on a second-order Costas loop implemented in discrete time. Let $T_{s}$ denote the input sampling period and $f_{s}=1/T_{s}$ the sampling frequency. The loop filter is realized in a state-space form, which is particularly amenable to parallel and pipelined FPGA implementation. The normalized loop bandwidth is defined as $B_{L}T_{s}$, where $B_{L}$ is the equivalent noise bandwidth of the Costas loop. Throughout this section, we analyze how the parallel architecture and its associated delays affect the maximum stable value of $B_{L}T_{s}$.

### 4.2. Definition of Parallel Equivalent Delay (PED)

In the proposed architecture, the Costas loop is implemented with a parallelization factor *N* and an internal processing clock frequency $f_{clk}$. In each input sampling period $T_{s}$, the loop processes *N* sub-iterations using *N* clock cycles of duration $T_{clk}=1/f_{clk}$. For a given input sampling rate, the effective parallelization factor is

(59) $$N=\frac{T_{s}}{T_{clk}}$$

Parallelism and pipelining introduce an additional “effective” delay into the feedback path of the loop. To relate this implementation-dependent delay to the continuous-time model, we define the parallel equivalent delay (PED) as the total additional group delay experienced by the feedback signal due to parallel architecture. There are two primary sources contributing to PED:

- Structural delay ($D_{struct}$) due to the fact that the loop update at time index *k* is based on samples that have passed through a chain of N−1 intermediate parallel stages;
- Computational delay ($D_{calc}$) due to the finite number of serial operations that must be performed within each sampling period, even when parallel hardware is available.

Let *S* denote the number of serial operations per input sample that cannot be fully overlapped in the algorithm realization. Under the assumption of a real-time implementation, the total PED can be expressed as

(60) $$D_{eq}=D_{struct}+D_{calc}=N-1+S\cdot \;N$$

Here, $D_{eq}$ is given in units of input sampling periods $T_{s}$. The term (N−1) models the structural delay associated with the parallel pipeline, while the term SN captures the cumulative effect of computational latency that scales with the parallelization factor *N*. This model for $D_{eq}$ will be used in the following subsections to quantify the impact of the architecture on stability and loop bandwidth.

### 4.3. Impact of PED on Loop Stability and Bandwidth

The open-loop transfer function for the parallelized system, $G_{N}\left(s\right)$, becomes:

(61) $$G_{N}\left(s\right)=K\frac{s+z}{s^{2}}\cdot e^{\left(-s{T_{s}D}_{eq}\right)}$$

To analyze the effect of the delay term $e^{\left(-s{T_{s}D}_{eq}\right)}$, we can use a first-order Padé approximation:

(62) $$e^{\left(-sT_{s}D_{eq}\right)}\approx \frac{1-s{T_{s}D}_{eq}/2}{1+s{T_{s}D}_{eq}/2}=-\frac{sT_{s}-2/D_{eq}}{sT_{s}+2/D_{eq}}$$

Substituting this approximation back into the transfer function:

(63) $$G_{N}\left(s\right)\approx -K\cdot \frac{s+z}{s^{2}}\cdot \frac{s-2/\left({T_{s}D}_{eq}\right)}{s+2/\left({T_{s}D}_{eq}\right)}$$

The introduction of this delay term fundamentally alters the system’s dynamics. It adds a stable pole in the LHP at $-2/\left({T_{s}D}_{eq}\right)$, but more importantly, it introduces an unstable zero in the right-half-plane (RHP) at $s=+2/\left({T_{s}D}_{eq}\right)$. The presence of the RHP zero dramatically affects stability. As the degree of parallelization (*N*) increases, the delay $D_{eq}$ becomes larger. This causes the RHP zero (at $2/{T_{s}D}_{eq}$) to move closer to the origin of the s-plane. As shown in Figure 4b, the root locus analysis shows that branches are now “pulled” towards this RHP zero. Consequently, the value of loop gain K at which the root locus crosses the imaginary axis (the threshold of instability) decreases as $D_{eq}$ increases.

In the frequency domain, the delay term $e^{\left(-j\omega *{T_{s}D}_{eq}\right)}$ introduces an additional negative phase shift of $-\omega {T_{s}D}_{eq}$ radians to the loop’s frequency response. This phase lag increases linearly with frequency ω. This phase lag directly erodes the loop’s phase margin, which is the primary indicator of its stability.

This degradation of phase margin leads to a critical performance limitation: for a given level of parallelism, there exists a maximum achievable loop bandwidth ($B_{Lmax}$) beyond which stability cannot be guaranteed. A robust feedback system typically requires a phase margin of at least 45°. The phase shift introduced by PED at the crossover frequency $\omega_{c}$ must not consume the entire available margin. By setting a limit on the maximum allowable phase degradation, there is a fundamental design constraint:

(64) $$\begin{array}{cc} B_{Lmax}T_{s} & \approx \frac{C}{D_{eq}} \end{array}$$

The constant *C* is an empirically validated design parameter that encapsulates the complexities of the loop’s stability requirements. Its value is determined by the trade-off between stability (phase margin) and the tolerable degradation from the ideal serial loop.

Equation (64) reveals the key impact of PED on loop performance: for a fixed sampling period $T_{s}$, the maximum stable normalized loop bandwidth is approximately inversely proportional to the equivalent normalized delay $D_{eq}$, which in turn is determined by the PED through Equation (60).

### 4.4. Throughput–Bandwidth Product (TBP) and the Stability Constant C

To summarize the trade-off between loop bandwidth and implementation delay in a compact form, we introduce the Throughput–Bandwidth Product (TBP). For a given loop architecture, the TBP is defined as

(65) $$\begin{array}{c} \mathrm{TBP}≜B_{Lmax}T_{s}\cdot D_{eq} \end{array}$$

Combining (64) and (65) yields

(66) $$\begin{array}{c} \mathrm{TBP}=\left(B_{Lmax}T_{s}\right)D_{eq}\approx C \end{array}$$

We therefore interpret *C* as a stability constant of the considered loop implementation: for a fixed loop order, loop filter structure, and phase-margin requirement, the TBP is expected to remain approximately constant and numerically equal to *C*.

In this context, the “throughput” aspect is implicitly captured by the parallelization factor *N* and the processing clock period $T_{clk}$, which together determine the PED and its normalized form $D_{\mathrm{eq}}$. The “bandwidth” aspect is quantified by the normalized maximum loop bandwidth $B_{Lmax}T_{s}$. The TBP relation (66) thus formalizes the intuitive statement that, for a given loop topology, any increase in PED (or equivalently in $D_{eq}$) must be compensated by a reduction in the achievable loop bandwidth to maintain stability.

### 4.5. Design Implications of PED and TBP

The PED and TBP analyses provide direct guidance for the design of parallel FPGA implementations of Costas loops and related feedback loops. For a fixed input sampling rate $1/T_{s}$ and a given throughput requirement, the designer mainly controls two architectural parameters: (a) the parallelization factor *N*, which influences the PED through both the structural delay and the computational delay terms in (60); (b) the internal processing clock frequency $f_{clk}=1/T_{clk}$, which determines how much computation can be performed within each sampling period without excessively increasing *N*.

From (60) and (64), it follows that increasing *N* while keeping $f_{clk}$ relatively low tends to increase the PED and its normalized counterpart $D_{eq}$, thereby reducing the maximum stable normalized loop bandwidth $B_{L,\max}T_{s}$. Conversely, for the same throughput target, operating the processing logic at a higher clock frequency allows the use of a smaller *N*, which decreases $D_{eq}$ and thus enables a larger achievable loop bandwidth. In practical terms, this leads to the following design guideline: For a given throughput constraint, loop-bandwidth performance is optimized by minimizing the PED, i.e., by operating at the highest feasible processing clock frequency and using the smallest degree of parallelism that still meets the throughput requirement.

From a design perspective, different values of *C* impose clear performance trade-offs: a larger *C* allows a higher normalized loop bandwidth $B_{L,\max}T_{s}$ for a given equivalent delay $D_{eq}$. This enables faster tracking and a wider effective capture range, but is typically associated with tighter phase margins and more aggressive transient responses (larger overshoot, shorter settling time). A smaller *C* corresponds to a more conservative design, with higher phase margin and smoother transients, but a lower achievable $B_{L,\max}T_{s}$ for the same $D_{eq}$. In practice, this reduces the maximum tolerable phase-noise dynamics or frequency offset for a fixed parallel architecture.

## 5. Simulation Results and Discussion

To validate the theoretical framework and evaluate the performance of the proposed state-space parallelization method, we conducted a series of comprehensive simulations. The method was applied to a Costas phase-locked loop (PLL) for carrier recovery in a coherent optical communication system. The simulation environment was configured with the following key parameters: the modulation format was Quadrature Phase-Shift Keying (QPSK) at a symbol rate of 10 Gbaud. A four-times oversampling ratio was employed, resulting in a sampling rate of 40 Gsps. Pulse shaping was performed using a Root-Raised Cosine (RRC) filter with a roll-off factor of 0.5. The channel was modeled with Additive White Gaussian Noise (AWGN), and laser phase noise was simulated as a Wiener process. For comparison, the performance of the classical open-loop Viterbi–Viterbi (VV) algorithm was also evaluated under identical conditions.

For a fundamental and standardized evaluation, system performance is measured in terms of the bit-energy-to-noise-power-spectral-density ratio (Eb/N0). This metric provides a normalized basis for comparison that is independent of specific system parameters such as modulation format, symbol rate, or channel bandwidth, facilitating a fair assessment against theoretical limits and other digital communication schemes.

For consistency, the OSNR measured in the optical experiments is converted to Eb/N0 using the following relation:

(67) $$\begin{array}{cc} \mathrm{OSNR} & =E_{b}/N_{0}+10\log_{10}\left(\frac{R_{s}\cdot m}{B_{ref}}\right) \end{array}$$

where m is the number of bits per symbol, $R_{s}$ is the symbol rate, and $B_{ref}$ is the optical reference bandwidth used in the OSNR measurement. In this system, with $R_{s}=10\;\mathrm{Gbaud}$, m=2(QPSK), and $B_{\mathrm{ref}}=0.1\;\mathrm{nm}\;(\approx$12.5 GHz), the conversion becomes:

(68) $$\begin{array}{c} \mathrm{OSNR}=E_{b}/N_{0}+2.04 \end{array}$$

Thus, all BER performance curves in this section are plotted against Eb/N0 to ensure comparability between simulation and experimental results, independent of modulation format, symbol rate, or measurement bandwidth.

### 5.1. Frequency Tracking Performance and Locking Behavior

**Figure 5:**
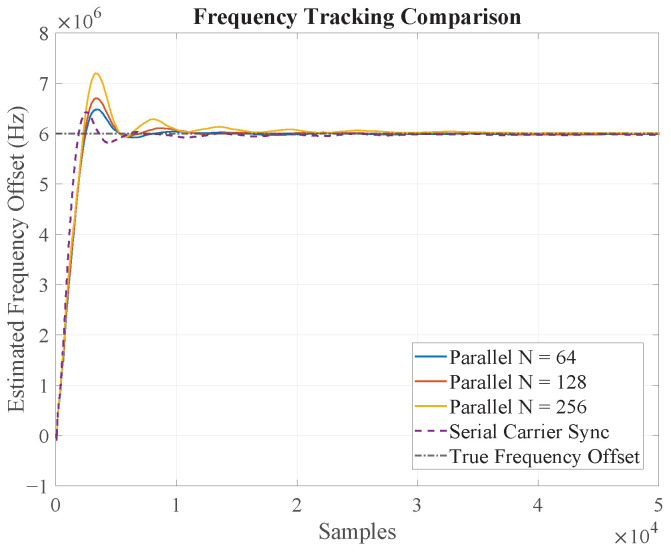
Dynamic frequency tracking performance of the proposed parallel Costas loops (*N* = 64, 128, 256) compared to the conventional serial implementation under a carrier frequency offset. All parallel structures demonstrate successful frequency acquisition and locking.

First, we verified the fundamental locking capability and dynamic tracking performance of the parallelized Costas loop. Figure 5 depicts the frequency tracking curves for the serial loop and the proposed parallel structures with *N* = 64, 128, and 256 under a fixed carrier frequency offset. The plots clearly demonstrate that all parallel configurations successfully acquire and lock onto the carrier frequency, exhibiting convergence behavior that is qualitatively similar to the serial counterpart.

For a more quantitative analysis, Table 1 summarizes the mean error and standard deviation of the estimated frequency after the loops have achieved a steady state. Several key observations can be made:

1. The mean frequency error is effectively compensated in all cases, indicating that the parallel structures maintain tracking accuracy. The small residual mean error is inherent to the loop’s operation and does not systematically increase with the parallelism factor *N*.
2. The standard deviation of the frequency estimate exhibits a slight, gradual increase as *N* grows. This is a crucial and expected result, consistent with our theoretical analysis. The increased parallelism leads to a larger equivalent loop delay ($D_{eq}$), which slightly degrades the loop’s noise-filtering characteristics, resulting in a marginally higher phase noise variance. Nevertheless, this degradation is graceful and well-controlled.

### 5.2. Validation of the Throughput–Bandwidth Product

A core contribution of our work is the establishment of a theoretical limit on the loop bandwidth, encapsulated by the “Throughput–Bandwidth Product”. To empirically validate this constraint, we determined the maximum stable loop bandwidth ($B_{L}$) and the corresponding maximum frequency acquisition range for different parallelism factors. The results, presented in Table 2, provide direct evidence supporting our analysis in Section 4.

There is a clear inverse relationship between the parallelism factor *N* and the maximum achievable loop bandwidth. For instance, increasing *N* from 64 to 256 reduces the maximum $B_{L}$ from 0.018 to 0.005. This directly translates to a reduced frequency acquisition range from ±35 MHz down to ±9 MHz. There is a clear inverse relationship between the parallelism factor *N* and the maximum achievable loop bandwidth. Specifically, the product $N\times B_{Lmax}T_{s}$ remains remarkably constant across the different configurations. For example, for *N* = 64, the product is 64×0.018=1.152, while for *N* = 256, it is 256×0.005=1.28. This empirically validates the existence of a constant Throughput–Bandwidth Product, which for our specific loop filter design is approximately 1.2. This fundamental trade-off provides a critical and quantifiable design guideline: achieving higher throughput via parallelism comes at the direct and predictable cost of reduced loop bandwidth, which in turn limits the system’s ability to track rapid phase variations or large carrier frequency offsets.

### 5.3. Bit Error Rate (BER) Performance

**Figure 6:**
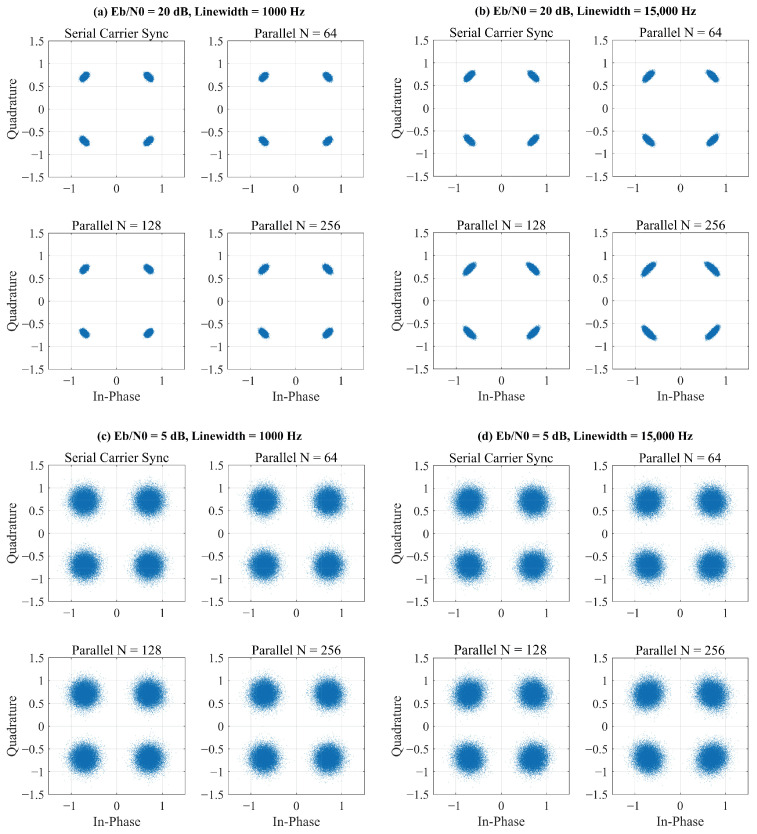
Comparison of received signal constellations after carrier recovery. The plots show the performance of the serial loop versus the proposed parallel loop at high Eb/N0 (**a**,**b**), low Eb/N0 (5 dB) (**c**,**d**), and with different laser phase noise, demonstrating robust carrier locking by the parallel structure.

To assess the impact of the parallel architecture on the end-to-end system fidelity, we evaluated the Bit Error Rate (BER) performance. Figure 6 shows the constellation diagrams after carrier recovery for both serial and parallel loops under various conditions. In all cases, the parallel loop successfully recovers the carrier, resulting in clean, well-defined constellation points.

**Figure 7:**
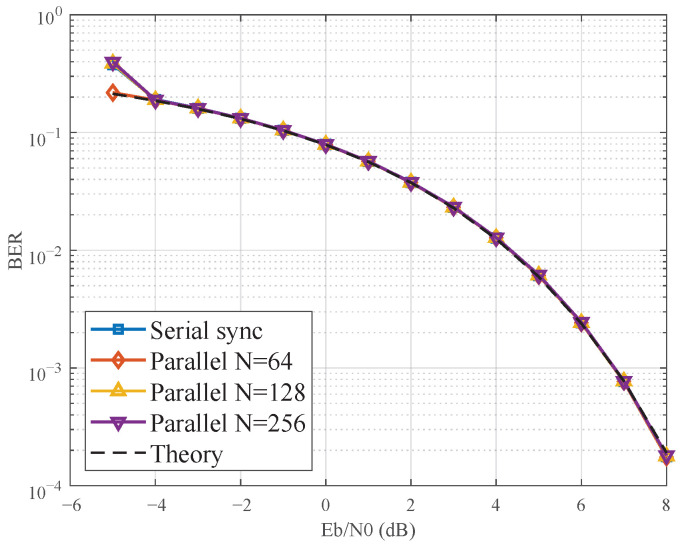
BER performance comparison between the serial Costas loop and the parallel implementations with *N* = 64, 128, and 256. The results show a minor and graceful performance degradation with increasing parallelism, confirming the high efficiency of the proposed method.

Figure 7 presents the BER performance curves. As shown, the parallelized loops achieve performance remarkably close to that of the ideal serial implementation. A minor power penalty is observable as *N* increases, which is a direct consequence of the slightly increased phase noise variance noted in Table 1. For instance, at a target BER of $10^{-3}$, the parallel loop with *N* = 256 incurs a power penalty of less than 0.5 dB compared to the serial loop. This confirms that our state-space method preserves the system’s performance integrity while enabling massive parallelism.

### 5.4. Comparison with the Open-Loop Viterbi–Viterbi Algorithm

**Figure 8:**
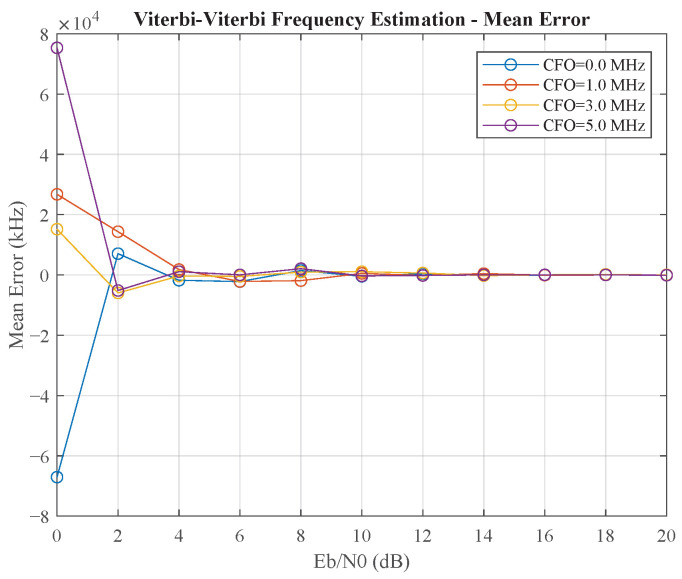
The mean estimation error (bias) of the Viterbi–Viterbi (VV) feedforward frequency estimator.

**Figure 9:**
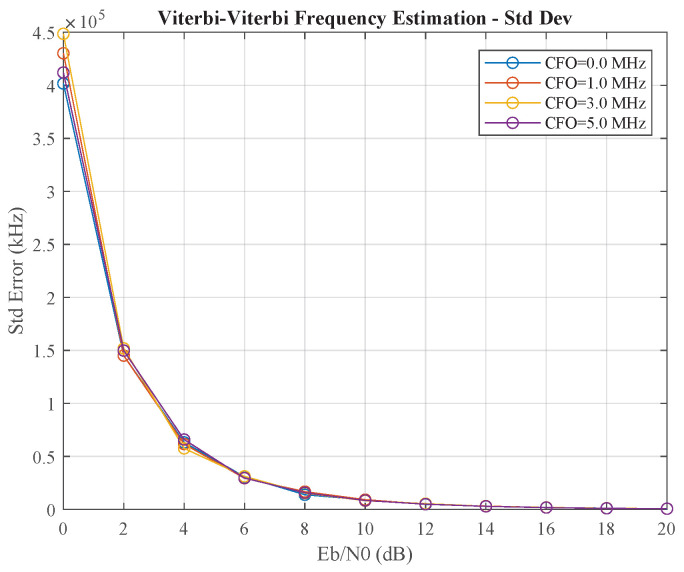
The standard deviation (variance) of the Viterbi–Viterbi (VV) feedforward frequency estimator.

Finally, we benchmarked our proposed parallel closed-loop method against the widely used open-loop Viterbi–Viterbi (VV) feedforward algorithm. Figure 8 and Figure 9 illustrate the performance of the VV frequency estimator. It shows a critical weakness: its accuracy degrades precipitously at low Eb/N0. Specifically, as shown in Figure 9, the standard deviation of the frequency estimate explodes to several hundred kHz for Eb/N0 below 8 dB, rendering the estimate unreliable. This contrasts sharply with the closed-loop method, which maintains a standard deviation below 1 kHz under the same conditions (as shown in Table 1). This performance collapse is characteristic of blind, non-data-aided feedforward estimators, as the underlying phase estimation relies on non-linear operations that amplify noise at low Eb/N0, leading to unreliable block-based estimates.

**Figure 10:**
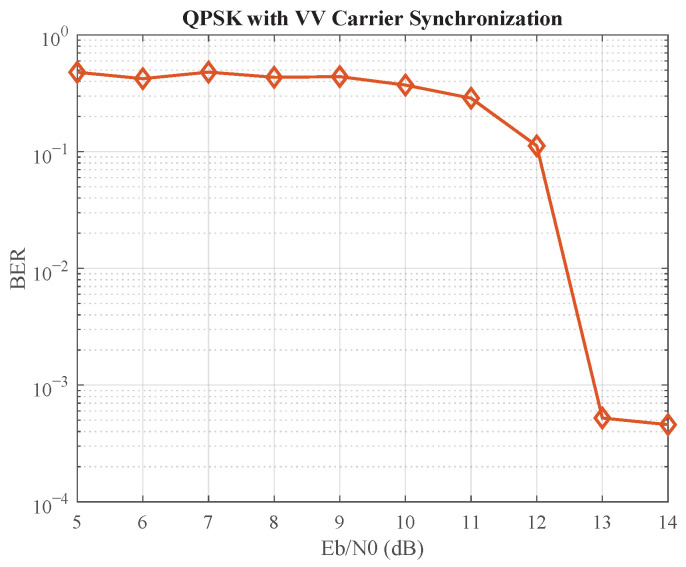
BER performance demonstrating the failure of the Viterbi–Viterbi algorithm at low Eb/N0. The high error floor makes it unsuitable for systems requiring robust performance in challenging channel conditions.

This poor estimation performance leads to a catastrophic failure at the system level, as shown by the high error floor in Figure 10. The system fails to achieve a BER below $10^{-2}$ even at an Eb/N0 of 13 dB. In stark contrast, our proposed parallel closed-loop structure (Figure 7) not only achieves a BER of $10^{-3}$ at an Eb/N0 of approximately 7 dB but also maintains robust locking even in these challenging conditions. This highlights a fundamental advantage of our method: by preserving the recursive nature of the feedback loop, it leverages the inherent noise-filtering and tracking capabilities that are crucial for reliable performance, making it vastly superior to open-loop estimators in channels characterized by low Eb/N0.

## 6. FPGA Implementation and Performance Analysis

To validate the feasibility and evaluate the hardware performance of the proposed parallel architecture, the carrier recovery loop was implemented on a Virtex UltraScale+ XCVU13P (FLGA2577-2i) FPGA. The design was synthesized and implemented using the Vivado 2022.1 Design Suite.

### 6.1. Experimental Setup and Resource Utilization

Unlike Block-FFT-based methods, which typically require the parallelism factor *N* to be a power of 2, the proposed state-space architecture supports arbitrary parallelism. This flexibility allows for the selection of an optimal parallelism factor (N=50) to precisely match the target line rate and clocking resources of the optical transmission system.

Table 3 summarizes the post-implementation resource utilization and timing analysis for the N=50 design under two different clock frequency constraints. At the target frequency of 312.5 MHz, the design achieves a throughput of 15.625 Gsps. The implementation is highly efficient, consuming only 6.5% of the available Look-Up Tables (LUTs) and 20.77% of the DSP slices, ensuring sufficient resources remain for other DSP modules.

### 6.2. Pipeline Depth and Verification of the Throughput–Bandwidth Product

Achieving high-frequency timing closure in recursive feedback loops necessitates the insertion of pipeline registers (*S*), which directly contribute to the loop latency. Based on the implementation results, the required pipeline depth *S* was modeled as a function of parallelism *N* and the target clock frequency ($F_{clk}$):

- For $F_{clk}=200\;\mathrm{MHz}$: $\;S\approx 14+\lceil \log_{2}N\rceil$;
- For $F_{clk}=312.5\;\mathrm{MHz}$: $\;S\approx 14+2\cdot \lceil \log_{2}N\rceil$.

It is important to note that these empirical formulas are contingent upon the specific process technology (16 nm FinFET) and speed grade (-2) of the target FPGA. While the logarithmic relationship with *N* is architectural, the baseline stages and the coefficient of the logarithmic term may vary across different FPGA families or synthesis strategies. The increase in the coefficient from 1 to 2 at higher frequencies reflects the necessity of additional retiming stages to break critical paths in the feedback loop.

To validate the theoretical stability analysis, we calculated the stability constant *C* using (60) and (66). Table 4 presents the measured performance metrics for different configurations. Despite the variations in parallelism (N=32 vs. N=50) and pipeline depth (S=24 vs. S=26), the product of the maximum stable bandwidth and total delay ($C=B_{Lmax}T_{s}\cdot D_{eq}$) exhibits remarkable consistency, converging to approximately 1.28. This empirical evidence strongly validates the proposed “Throughput–Bandwidth Product (TBP)” metric as a reliable predictor of system stability.

While the proposed architecture successfully achieves high throughput, the experimental results highlight a fundamental trade-off inherent to parallel feedback systems. As observed in the N=50 implementation, increasing the clock frequency from 200 MHz to 312.5 MHz necessitates an increase in pipeline stages from 20 to 26. This extension of the feedback path imposes a penalty on the loop latency, causing the maximum stable bandwidth ($B_{Lmax}T_{s}$) to degrade from $1.2\times 10^{-3}$ to $0.95\times 10^{-3}$.

Consequently, the frequency acquisition range is reduced from ±2.1 MHz to ±1.8 MHz. This phenomenon indicates that throughput enhancement via deep pipelining is constrained by the feedback latency bottleneck. For ultra-high-speed optical coherent receivers, this implies that the tracking capability for fast-varying phase noise is strictly bounded. Therefore, the proposed TBP metric serves as a critical design guideline, allowing engineers to determine the optimal operating point between throughput requirements and phase tracking performance prior to hardware implementation.

## 7. Conclusions

This paper presents a unified state-space-based framework for parallelizing both feedforward and feedback DSP algorithms in coherent optical receivers. By mapping serial algorithms into an equivalent MIMO state-space representation, the proposed method systematically derives parallel architectures using only matrix operations, eliminating ad hoc, algorithm-specific derivations. The framework is applicable to a broad class of FIR/IIR filters and tracking loops, and guarantees exact serial equivalence at the algorithmic level.

A key theoretical contribution of this work is the identification and analysis of the “parallel equivalent delay” (PED), an inherent latency in any parallel feedback architecture. Our analysis revealed that PED, composed of structural and computational delays, introduces a right-half-plane zero into the loop’s transfer function, fundamentally limiting its stability. This analysis led to the definition of a Throughput–Bandwidth Product (TBP), summarized by the relation (64), where $D_{eq}$ is the total normalized loop delay. The corresponding stability constant $C=B_{Lmax}T_{s}\cdot D_{eq}$ was found to be approximately 1.28 for the considered second-order Costas loop and implementation. This constant thus provided a practical design metric that linked parallelism, hardware latency, and loop dynamics.

The proposed framework was validated through the design of a highly parallel Costas carrier recovery loop. Simulations confirmed that the parallel loops retained the locking behavior and BER performance of the serial loop, with only a minor and graceful performance degradation as the parallelization factor increased. Compared with a classical Viterbi–Viterbi feedforward estimator, the parallel Costas loop exhibited dramatically improved robustness at low Eb/N0, avoiding the severe error floors observed for VV.

Finally, a 50-way parallel Costas loop was implemented on a AMD XCVU13P FPGA, achieving 15.625 Gsps at 312.5 MHz with less than 7% LUT utilization. Measured maximum stable loop bandwidths across different *N*, clock frequencies, and pipeline depths confirmed this.

Future work will extend the state-space parallelization and PED analysis to higher-order carrier recovery loops, adaptive equalizers, and timing recovery architectures, and investigate active PED compensation strategies in ultra-high-parallelism regimes. 

## Figures and Tables

**Table 1:** Steady-state frequency estimation error and standard deviation for serial and parallel Costas loops.

*N*	Mean Error (Hz)	Standard Deviation (Hz)
serial	−2232.65	825.18
64	−1533.57	847.40
128	−851.93	860.05
256	450.04	1040.94

**Table 2:** Maximum achievable loop bandwidth ($B_{\mathrm{Lmax}}$) and corresponding frequency acquisition range for different parallelism factors (*N*).

N	${\mathrm{BL}}_{\max}$	Max Frequency Offset
64	0.018	±35 MHz
128	0.010	±18 MHz
256	0.005	±9 MHz

**Table 3:** FPGA resource utilization and timing analysis (N=50 on XCVU13P).

Resource Type	Available	Utilization (200 MHz)	Utilization % (200 MHz)	Utilization (312.5 MHz)	Utilization % (312.5 MHz)
LUT	1,728,000	92,283	5.34%	112,243	6.50%
FF	3,456,000	133,213	3.85%	222,673	6.44%
BRAM	2688	25	0.93%	25	0.93%
DSP	12,288	2552	20.77%	2552	20.77%
WNS * (ns)	-	0.014	(Met)	0.006	(Met)

* Note: WNS denotes Worst Negative Slack. A positive WNS indicates successful timing closure. ns: not significant

**Table 4:** Throughput–Bandwidth trade-off verification.

Parallelism (N)	Clock Freq (MHz)	Throughput (Gsps)	Pipeline Stages (S)	Total Latency ($D_{\mathrm{eq}}$)	Max Stable BW ($B_{\mathrm{Lmax}}T_{s}$)	Max Freq Offset (MHz)	Stability Constant (*C*)
32	312.5	10.000	24	799	$1.60\times 10^{-3}$	±2.9	1.278
50	312.5	15.625	26	1349	$0.95\times 10^{-3}$	±1.8	1.281
50	200.0	10.000	20	1049	$1.22\times 10^{-3}$	±2.1	1.280

## Data Availability

The raw data supporting the conclusions of this article will be made available by the authors on request.
